# Phenotype of autosomal dominant Alport syndrome with a likely pathogenic heterozygous variant in the *COL4A3* gene (*Gly366Arg*) and incidental teratozoospermia: A case report

**DOI:** 10.23938/ASSN.1158

**Published:** 2026-04-20

**Authors:** Vicente Martín Moreno, Amanda Martín Fernández, Asunción García Mateos, Jorge Hurtado Gallar, Cristina Angulo García, Raquel Sánchez-Redondo Mayordomo

**Affiliations:** 1 Centro de Salud de Orcasitas Servicio Madrileño de Salud Madrid España; 2 Instituto de Investigación Hospital Doce de Octubre Madrid España; 3 Polibea Concierto Madrid España

**Keywords:** Alport Syndrome, COL4A3, Chronic Kidney Disease, Teratozoospermia, Microhematuria, Síndrome de Alport, Insuficiencia Renal Crónica, COL4A3, Teratozoospermia, Microhematuria

## Abstract

Autosomal dominant Alport syndrome (ADAS) is the least common form of Alport syndrome, the second most common monogenic cause of chronic kidney disease after autosomal dominant polycystic kidney disease.

We report a 14-year follow-up of a patient with ADAS carrying a likely pathogenic heterozygous variant in *COL4A3* (*Gly366Arg* substitution). The disease initially presented with microhematuria and no renal impairment on analysis or ultrasound. The patient progressively developed bilateral renal cysts and renal failure -a classic renal pattern of the syndrome- and bilateral hearing loss. Incidentally, infertility study (had not children) showed teratozoospermia and elevated levels of luteinizing hormone and progesterone, hormones associated with spermatogenesis. Long-term follow-up of rare variants of Alport syndrome helps refine genotype-phenotype correlations and disease progression. The incidental finding of teratozoospermia raises the possibility of an etiological association, that warrants further investigation as α3(IV) collagen chains involved in spermatogenesis are present in seminiferous tubules.

## RESUMEN

El síndrome de Alport autosómico dominante (ADAS) es la forma menos común del síndrome de Alport, que es la segunda causa monogénica de enfermedad renal crónica tras la enfermedad renal poliquística autosómica dominante.

Presentamos el fenotipo observado en el seguimiento durante 14 años de un paciente con ADAS con variante probablemente patogénica en *COL4A3* (sustitución *Gly366Arg*), que debutó con microhematuria, sin afectación renal analítica o ecográfica. El paciente desarrolló progresivamente quistes renales bilaterales e insuficiencia renal, patrón renal clásico del síndrome de Alport, e hipoacusia bilateral. Incidentalmente, el estudio de infertilidad (no tuvo hijos) mostró teratozoospermia e incremento de hormona luteinizante y progesterona, hormonas asociadas con la espermatogénesis. El seguimiento longitudinal de variantes raras del síndrome de Alport permite conocer sus fenotipos y patogenicidad. El hallazgo incidental de teratozoospermia abre un debate etiológico -que futuros estudios deberán abordar- al presentar los túbulos seminíferos cadenas *α3(IV)*, involucradas en la espermatogénesis.

## INTRODUCTION

Microhematuria, defined by the presence of three or more red blood cells per high-power field in a urine sample^1^^,^^2^, is observed in 4-5% of urinalyses^3^, with a prevalence ranging from 2 to 34% depending on the studied population and associated comorbidities^1^^,^^3^^,^^4^.

Among the recognized causes of microhematuria is Alport syndrome, a rare genetic disorder listed in Orphanet under descriptor 63. It is the second most common monogenic cause of chronic kidney disease^5^ and may be inherited in autosomal recessive, autosomal dominant, or X-linked forms, with each pattern influencing clinical presentation and prognosis^6^^,^^7^. The syndrome can present at any age, and its true global prevalence remains unknown. Regarding subtype distribution, recent studies suggest that the autosomal dominant form (ADAS) may be more frequent (68%)^8^. Moreover, more than 5,000 pathogenic variants have been described, reflecting marked phenotypic heterogeneity^9^.

Its etiology is associated with pathogenic variants in the genes encoding the alpha (*α*) chains of type IV collagen of the glomerular basement membrane, specifically *COL4A3*, *COL4A4* and *COL4A5*^6^^,^^7^. There are six types of *α* chains (*α1* to *α6*), forming three protomers: among them, the *α3.α4.α5(IV)* protomer is associated with Alport syndrome and is present in the glomerular, cochlear, and ocular basement membranes. The pathogenic role of the *α3* pathogenic variant (*COL4A3*), as in the presented case, remains under investigation^6^.

In this context, reporting cases with longitudinal follow-up contributes not only to the characterization of variant-associated phenotypes, but also to a better understanding of their clinical relevance. This is particularly important in rare diseases, where such evidence is essential for refining prognostic assessment and evaluating therapeutic outcomes.

## CASE DESCRIPTION

A 55-year-old male patient was diagnosed in 2011 with microhematuria (3-5 red blood cells per high-power field) on routine analysis, confirmed in a second sample, without anemia, renal impairment, or elevated prostate-specific antigen. He had no history of reno-ureteral colic, lower urinary tract symptoms, hypertension, diabetes, or use of nephrotoxic or hematuria-associated medications. He was an ex-smoker (quit in 2004). He had no children. He reported regular physical activity (gym training three times per week) due to his occupation as a security guard. His weight was 71 kg, height 168 cm, and body mass index 25.16 kg/m^2^.

Renal ultrasound was performed due to microhematuria and a family history of kidney disease (two cousins with kidney transplantation of unknown etiology). It revealed a 1 cm bladder diverticulum, with normal seminal vesicles (Table 1). Urine cytology was negative for malignant cells. Given the persistence of microhematuria and a family history, clinical follow-up was recommended.

During follow-up, the patient was diagnosed with hypertension in 2013 (mean blood pressure 143/78 mm Hg) and started on enalapril 5 mg twice daily. In 2014, due to suboptimal systolic control (140-150 mm Hg), the dose was increased to 10 mg twice daily. After one year of adequate control, treatment was reduced to 5 mg twice daily.

**Figure 1:**
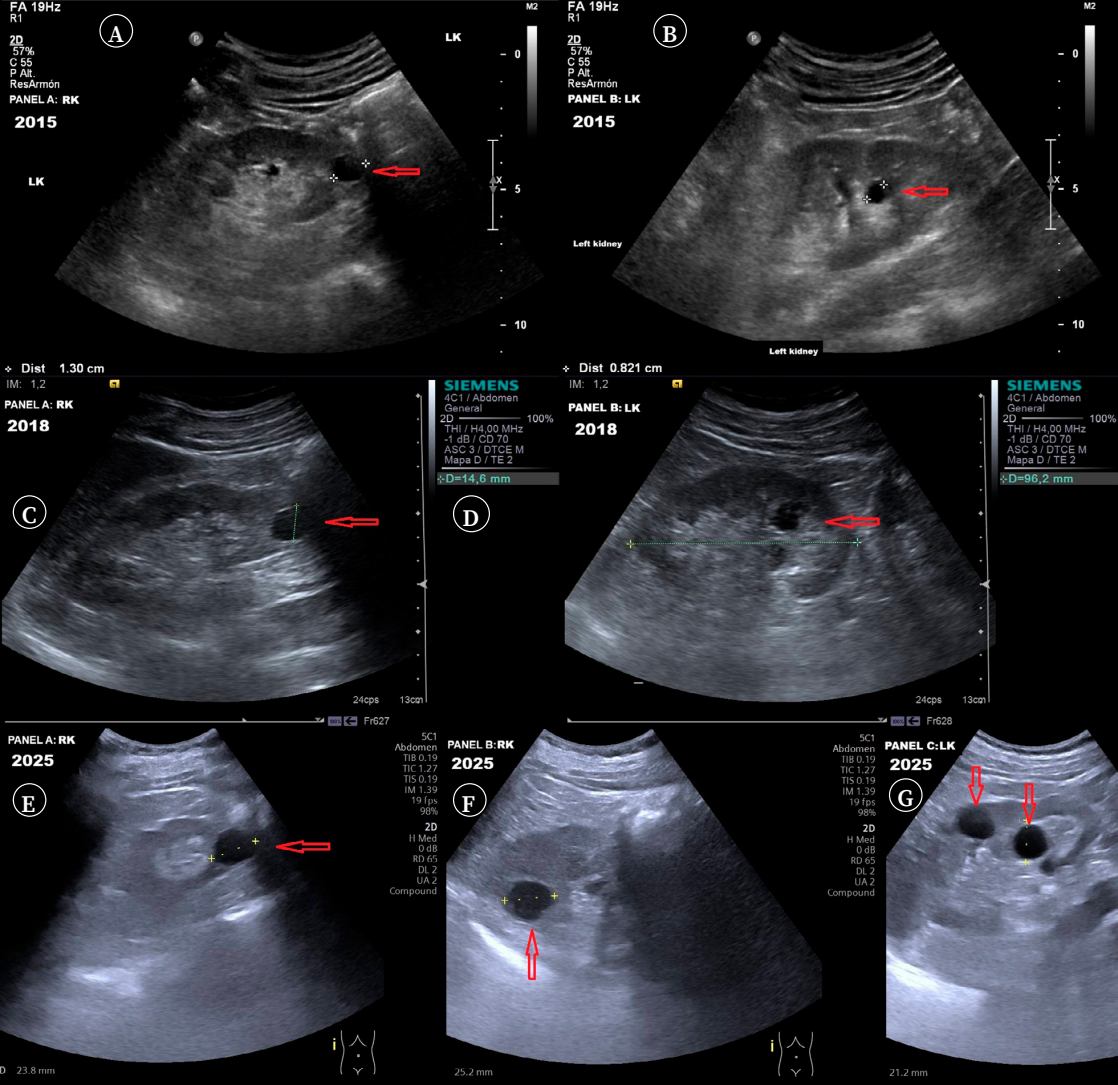
Changes observed in the urinary system during the period 2015-2025 by ultrasound. **A, B**. Small bilateral cortical cysts and sinus cysts in the left kidney (2015). **C, D**. 2018. Bilateral cortical cysts, the largest measuring 15 mm in the lower pole of the right kidney (C) and left sinus cysts measuring up to 16 mm (D). **E, F.** 2025. Right kidney: cortical cysts measuring 21 to 25 mm. **G**. 2025. Left kidney sinus cyst measuring 21 mm and cortical cyst measuring 28 mm. Red arrows: location of the renal cysts.

Microhematuria persisted, with progressive renal ultrasound changes (Table 1, Fig. 1). While the initial ultrasound in 2011 was normal, by 2015 bilateral small cortical cysts and a subcentimetric sinus cyst in the left kidney were detected. In 2016, sinus cysts became bilateral, although renal function remained normal at that time.

Renal cysts progressively increased, reaching 15mm in the right cortex and 16mm in the left renal sinus in 2018 (Fig. 1). At this stage, renal function impairment appeared, with an estimated glomerular filtration rate (eGFR) of 60 mL/min/1.73 m^2^ and elevated creatinine (Table 1).

Further deterioration was observed in 2019 (eGFR 55 mL/min/1.73 m^2^, creatinine 1.36 mg/dL), prompting genetic testing. Whole-exome sequencing (xGen Exome Panel v1.0) targeting nephronophthisis and polycystic kidney disease genes identified a heterozygous c.1096G>A (p.Gly366Arg) variant in *COL4A3* (NM_000091.4), resulting in substitution of glycine for arginine at position 366 within a collagenous domain of the α3 chain. This variant is consistent with autosomal dominant inheritance and supports the diagnosis of ADAS. Autoimmune screening, including anti-DNA, antinuclear, SSA/Ro, SSA/Ro52, SSA/Ro60, SSB/La, RNP 68, centromere protein B, TOPO-I/SCL-70, JO-1/HRS, ribosomal P, Sm, SM-RNP, chromatin, myeloperoxidase, proteinase 3, and anti-glomerular basement membrane antibodies, was negative. Immunoglobulin levels (IgA 340 mg/dL, IgG 1,290 mg/dL, IgM 121 mg/dL) and complement levels (C3 86.3 mg/dL, C4 21 mg/dL) were within normal ranges, as was BP (133/57 mm Hg).

**Figure 2:**
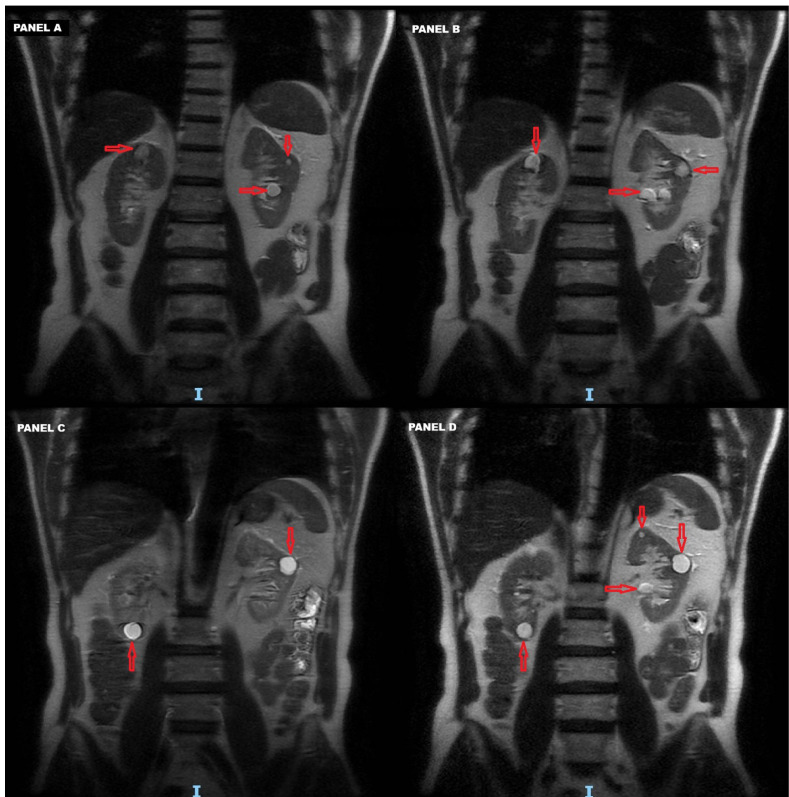
Abdominal magnetic resonance imaging performed in 2021 showing bilateral cortical and medullary cysts at renal level. **A-D**. Serial sections showing the progressive presence of renal cysts in a depth-wise scan. Craniocaudal description: Right kidney: upper pole 17 mm, middle third 7 mm, lower pole 17 mm. Left kidney: upper pole 6 mm (x2), middle third 17 mm and 6 mm, lower third two sinus cysts of 18 mm and 16 mm. Red arrows: location of the renal cysts.

The renal disease followed a progressive course, with abdominal MRI in 2021 showing further enlargement of both cortical and sinus renal cysts (Table 1, Fig. 2).

Between 2023 and 2024, renal function continued to decline (creatinine 1.44 mg/dL in May 2023, 1.48 mg/dL in November 2023, and 1.53 mg/dL in April 2024; eGFR 46 mL/min/1.73 m^2^), without proteinuria or hematuria, and BP of 100/60 mmHg). Treatment was switched to lisinopril 5 mg twice daily.

At present (2025), the patient is asymptomatic from a renal perspective. He presents bilateral hearing loss at 4,000 Hz, which, although not typical of ADAS, may represent an early manifestation. Ophthalmological evaluations in 2022 and 2025, including fundus examination, were normal. He remains physically active (gym training three times per week). Current weight is 68.5 kg, height 168 cm, BMI 24.27 kg/m^2^. Renal function continues to decline (eGFR 46 mL/min/1.73 m^2^, creatinine 1.51 mg/dL), with normal alpha-1 antitrypsin levels (143 mg/dL; reference: 90-200), no anemia, microalbuminuria, or prostate-specific antigen abnormalities. Ultrasound shows progressive enlargement of renal cysts, the largest measuring 21-25 mm in the right kidney and 23-28 mm in the left kidney. He continues lisinopril 10 mg twice daily, with current BP of 110/66 mmHg.

Regarding infertility evaluation, physical examination of the testes was normal, with no evidence of hypoplasia, nodules, absence, or varicocele. Hormonal analyses (Table 1) showed elevated LH (9.82 IU/L) and progesterone (0.39 ng/mL), hormones related to testicular function and spermatogenesis regulation. Semen analysis, performed after six days of abstinence, showed low ejaculate volume and teratozoospermia. Only 1% of spermatozoa exhibited normal morphology. However, sperm concentration, vitality, and motility were within normal ranges.

**Table 1:** Chronological follow-up and studies performed

ALPORT SYNDROME								
Classic model with renal disease								
*Analytical data in relation to renal function*								
Parameter	2011	2014	2016	2018	2020	2022	2024	2025
Hematuria (erythrocytes/field)	5	6	2	5	2	4	0	0
GFR (mL/min/1.73m^2^)	-	-	-	60	54	54	45	46
Creatinine (mg/dL)	N^11^	1.18	1.16	1,27	1.37	1.36	1.55	1.51
Albuminuria (mg/dL)	-	0.1	0.3	-	0.6	0.2	0.13	0.33
Proteinuria (mg/dL)	N	0	0	0	0	0	0	0
PTH (pg/mL)	-	17.5	59.7	47.7	-	39.4	73.9	69.6
Glucose (mg/dL)	N	76	82	76	87	85	85	88
Glycated hemoglobin (%)	N	4.9	5.1	5.1	-	5.4	5.3	5.3
Hemoglobin (g/dL)	16.2	14.3	15	15.9	15.1	15.1	14.4	14.7
PSA (ng/mL)	0.49	0.35	0,64	1.28	-	0,59	0.63	-
*Imaging studies on renal involvement, extended to bladder and prostate*								
ECOGRAPHY	2011		2015		2016		2018	
	- No renal cysts		- Small bilateral cortical cysts, subcentimeter RI sinus cyst		- Bilateral cortical and sinus cysts		- Enlargement of bilateral cortical and sinus cysts	
	- Bladder diverticulum 1 cm		- Normal bladder		- Bladder wall trabeculated with a small diverticulum on the left side		- Bladder wall with irregular thickening and multiple diverticula	
	- Prostate 47x19x2mm, weight 10 g		- Prostate 37x30x36 mm, volume 21 cc		- Prostate volume 20.2 cc		- Prostate volume 33 cc	
MAGNETIC RESONANCE IMAGING	2021							
	Both kidneys present simple cortical cysts, which described craniocaudally are:							
	- Right kidney: upper pole 17 mm, middle third 7 mm, lower pole 17 mm							
	- Left kidney: upper pole 6 mm (x2), middle third two, 17 mm and 6 mm							
	- Sinus cysts in the lower third of 18 mm and 16 mm							
*Infertility study*								
SEMINOGRAM	Volume: 0.5 mL (standard ≥1.4)							
	Sperm concentration: 168.25 millon/mL							
	Vitality index (74%) and motility (progressive 47%, total 54%) normal, but only 1% of spermatozoa had normal morphology							
HORMONAL STUDY	Testosterone: 670 ng/dL					Progesterone: 0.39 ng/mL		
	Prolactin: 19 ng/mL					Estradiol: 27.6 pg/mL		
	FSH: 6.86 IU/L					Cortisol: 19.1 µg/dL		
	LH: 9.82 IU/L					IGF-1: 180.8 ng/mL		
	Androstenodione: 2.39 ng/mL					17-OH-progesterone: 1.59 ng/mL		
	DHEA-S: 171 µg/dL					Folic acid: 5 ng/mL		

GFR: glomerular filtration rate; PTH: parathyroid hormone; PSA: prostate specific antigen; FSH: follicle stimulating hormone; HL: luteinizing hormone; DHEA-S: dehydroepiandrosterone sulfate; IGF-1: insulin-like growth factor.

Normal ranges: hematuria: <3; GFR: ≥60 mL/min/1.73m^2^; Creatinine: <1.20; albuminuria >3.0; proteinuria: 0 mg/dL; PTH: 17.3-74.1; glucose: 70-110; glycated hemoglobin: 4-5.6; hemoglobin: 13-16.8 g/dL; PSA: 0.01-4.1; testosterone: 193-740; prolactin: 4-15.2; FSH: 1.5-12.4; LH: 1.7-8.6; androstenodione: 0.6-2.7; DHEA-S: 33.6-249; progesterone: ≤0.15; estradiol: 11.5-43.2; cortisol: 6-18; IGF-1: 80-220; 17-OH-progesterone: <2.2; folic acid: 3.9-26.8.

## DISCUSSION

Persistent microhematuria and a family history of kidney disease raised clinical suspicion, further supported by progressive renal function decline^1^^-^^3^^,^^5^ and the development of renal cysts during follow-up. These findings justified genetic testing^7^, which confirmed the diagnosis of ADAS^5^^,^^7^. The patient subsequently received genetic counseling regarding family screening^7^^-^^9^. He had no children and his two sisters declined genetic testing.

As in this case, patients with ADAS are often asymptomatic, with a notable dissociation between clinical presentation and the severity of findings on diagnostic evaluation^10^. Auditory involvement is also less frequent in this subtype^5^^-^^7^^,^^10^.

The phenotype observed, associated with the likely pathogenic heterozygous *COL4A3* c.1096G>A (p.Gly366Arg) variant, included hematuria, progressive development of cortical and medullary renal cysts, and gradual renal function decline, without proteinuria or other signs of nephritis. Although no ocular abnormalities were detected, the presence of bilateral hearing loss -while not typical of ADAS- may represent an early manifestation of the disease spectrum. This phenotypic presentation, not previously described for this variant, expands the spectrum associated with COL4A3 and complements findings from other longitudinal studies with similar follow-up durations^11^.

In terms of prognosis, ADAS generally follows a milder course than other forms of Alport syndrome, with fewer than 3% of patients requiring dialysis by 80 years. Population-based data, such as those from the UK Biobank^12^, suggest that *COL4A3* variants may be more prevalent than previously recognized (approximately 1 in 106 individuals)^11^^,^^13^, with many carriers remaining asymptomatic. However, heterozygous pathogenic variants, particularly those affecting collagenous domains, are associated with and increased risk of hematuria, chronic kidney disease, and renal failure^11^, as observed in this case.

Clinically, management of ADAS is supportive and renoprotective. Current guidelines recommend early blockade of the renin-angiotensin system^9^ to delay progression to kidney failure. Disease progression appears more frequent in males^14^, and in individuals who develop microalbuminuria at an early age^15^. Substitutions of glycine residues, such as *Gly* → *Arg*, are also associated with a higher likelihood of disease expression^12^. Renal cysts are more commonly observed in older patients with ADAS and microalbuminuria^16^, and may contribute to a clinical pattern resembling nephroangiosclerosis, particularly in males^17^, although some authors consider cystic changes and ADAS as distinct entities^17^.

Finally, the clinical variability of ADAS, even within the same family, variable penetrance of the same mutation, and the dissociation between genotype and phenotype contribute to diagnostic challenges and likely underdiagnosis^9^^,^^12^.

During follow-up, severe isolated teratozoospermia was identified incidentally, an uncommon condition (<5%) that may explain the patient´s infertility. The patient and his partner had not previously sought medical evaluation for infertility. No relevant history of abortions or surgery, toxic exposure, contraceptives, medication use, or endocrine or neoplastic disorders were identified. Age may be a contributing factor, as the risk and severity of sperm morphological abnormalities increase after the age of 40^18^. Hormonal evaluation revealed mild elevation in luteinizing hormone and progesterone, while other parameters were within normal ranges. Chronic kidney disease itself may also contribute to impaired spermatogenesis.

In the absence of a clear etiology, a potential association with Alport syndrome should be considered. Experimental animal studies have shown that *COL4A3-*derived chains are present in the basement membrane of seminiferous tubules and generate biologically active collagen fragments, such as NC1, involved in the regulation of spermatogenesis^19^^,^^20^. Disruption of these pathways may affect cytoskeletal organization of F-actin and microtubule networks through mechanisms involving mTORC1 / p-rpS6 / p-Akt1 / 2 signaling, thereby impairing spermatogenesis^20^^-^^22^. Evidence from human studies also suggests a role for type IV collagen in testicular function, with reduced activity associated with impaired sperm motility^23^^,^^24^. Other studies report that the NC1 mutation itself could be associated with Alport syndrome^25^.

The etiology of isolated teratozoospermia remains uncertain^26^. In this context, the present finding should be considered hypothesis- generating^13^, supported by biological plausibility from animal models and emerging human data. Further studies in larger cohorts are required to determine whether this association represents coincidence, a modifying factor, or a true extension of the phenotypic spectrum of ADAS.

Finally, the age of the patient and his partner, together with their decision not to pursue parenthood, precluded further reproductive investigations, including repeat semen analysis, testicular biopsy, or genetic analysis of sperm. In addition, the refusal of family members to undergo genetic testing limited the assessment of intrafamilial variability and penetrance.

In conclusion, ADAS remains an underrecognized condition with substantial phenotypic variability. This 14-year longitudinal follow-up highlights progressive renal involvement and suggests the possibility of extrarenal manifestations beyond the classical triad. Although causality cannot be established, the coexistence of genetically confirmed ADAS and isolated teratozoospermia raises the possibility of an expanded clinical spectrum. Further studies in larger cohorts are required to clarify whether reproductive involvement represents a coincidental finding or a true phenotypic feature of the disease.

## Data Availability

The data of this clinical report is available on request to the corresponding author.
